# Antioxidant Properties of Egg White Hydrolysate Prevent Mercury-Induced Vascular Damage in Resistance Arteries

**DOI:** 10.3389/fphys.2020.595767

**Published:** 2020-11-20

**Authors:** Alyne Goulart Escobar, Danize Aparecida Rizzetti, Janaina Trindade Piagette, Franck Maciel Peçanha, Dalton Valentim Vassallo, Marta Miguel, Giulia Alessandra Wiggers

**Affiliations:** ^1^Graduate Program in Biochemistry and Multicentric Graduate Program in Physiological Sciences, Universidade Federal do Pampa, Uruguaiana, Brazil; ^2^Department of Physiological Sciences, Universidade Federal do Espírito Santo and School of Medicine of Santa Casa de Misericórdia (EMESCAM), Vitória, Brazil; ^3^Bioactivity and Food Analysis Department, Instituto de Investigación en Ciencias de la Alimentación, Campus Universitario de Cantoblanco, Madrid, Spain

**Keywords:** egg white hydrolysate, antioxidant properties, mercury, mesenteric resistance arteries, vascular damage, nitric oxide, oxidative stress

## Abstract

**Aim:** We investigated the antioxidant protective power of egg white hydrolysate (EWH) against the vascular damage induced by mercury chloride (HgCl_2_) exposure in resistance arteries.

**Methods:** Male Wistar rats received for 60 days: (I) intramuscular injections (i.m.) of saline and tap water by gavage – Untreated group; (II) 4.6 μg/kg of HgCl_2_ i.m. for the first dose and subsequent doses of 0.07 μg/kg/day and tap water by gavage – HgCl_2_ group; (III) saline i.m. and 1 g/kg/day of EWH by gavage – EWH group, or (IV) the combination of the HgCl_2_ i.m. and EWH by gavage – EWH + HgCl_2_ group. Blood pressure (BP) was indirectly measured and dose-response curves to acetylcholine (ACh), sodium nitroprusside (SNP), and noradrenaline (NE) were assessed in mesenteric resistance arteries (MRA), as *in situ* production of superoxide anion, nitric oxide (NO) release, vascular reactive oxygen species (ROS), lipid peroxidation, and antioxidant status.

**Results:** Egg white hydrolysate prevented the elevation in BP and the vascular dysfunction after HgCl_2_ exposure; restored the NO-mediated endothelial modulation and inhibited the oxidative stress and inflammatory pathways induced by HgCl_2_.

**Conclusion:** Egg white hydrolysate seems to be a useful functional food to prevent HgCl_2_-induced vascular toxic effects in MRA.

## Introduction

The imbalance between the excessive formation of reactive oxygen species (ROS) and limited antioxidant defenses is one of the most harmful mechanisms that induce deleterious cardiovascular effects (Liguori et al., 2018). Oxidative damage has been implicated in vascular injury and endothelial dysfunction (Halliwell, 2007), a proliferation of vascular smooth muscle cells (VSMC), increase in vascular tone, migration of inflammatory mediators, vascular remodeling (Szasz et al., 2007), and hypertension (Touyz and Briones, 2011; Montezano and Touyz, 2014; Montezano et al., 2015; Incalza et al., 2018).

Several studies point out the beneficial effects of consuming different natural antioxidant compounds and bioactive food ingredients, such as protein-derived peptides against oxidative imbalance (Serafini and Peluso, 2016; Peluso and Serafini, 2017; Chikara et al., 2018). Bioactive peptides from egg white hydrolysate (EWH) have shown important antioxidant properties, preventing dysfunction in hypertensive and obese experimental models (Manso et al., 2008; Garces-Rimon et al., 2019). We propose to use EWH obtained after hydrolysis of hen egg white with pepsin for 8 h, which has previously demonstrated antioxidant, anti-inflammatory, and angiotensin-converting enzyme (ACE) inhibitory properties (Miguel et al., 2004; Moreno-Fernandez et al., 2018a,b).

Metals, such as mercury (Hg), are dangerous pollutants in the ecosystem, found in different physical and chemical forms. Their toxic effects are dose and time-dependent and can be characterized as a risk factor for the development of cardiovascular diseases by the promotion of oxidative stress (Genchi et al., 2017). Hg participates in the Fenton reaction, increasing ROS production and also leading to the depletion of important antioxidant enzymes due to their affinity for the sulfhydryl radicals (Valko et al., 2006; Su et al., 2008).

Studies have been shown that 30-day exposure to a low concentration of mercury chloride (HgCl_2_) induces oxidative stress and activation of cyclooxygenase (COX) and angiotensin II pathways, promoting vascular changes, such as endothelial dysfunction and increased reactivity (Wiggers et al., 2008; Peçanha et al., 2010). It has been found that EWH improves the Hg-induced damage in parameters related to memory deficits (Rizzetti et al., 2016a), peripheral nervous disorders (Rizzetti et al., 2016b), male reproductive dysfunction (Rizzetti et al., 2017b), and conductance arteries injury (Rizzetti et al., 2017a). In aorta of long-term Hg-exposed rats, EWH prevented the high vascular reactivity and endothelial dysfunction promoted by the metal. However, small arteries, such as resistance mesenteric arteries (MRA), represent the primary vessels that are involved in the regulation of arterial blood pressure (BP) as well as blood flow within the organ.

Thus, we investigate whether the antioxidant properties of EWH have a protective power against vascular damage caused by exposure to HgCl_2_ in MRA and the underlying pathways involved.

## Materials and Methods

### Animals and Experimental Groups

The experiments were conducted in compliance with the Principles of Laboratory Animal Care (National Institutes of Health publication 80–23, revised 1996) and in agreement with the guidelines by the Brazilian Societies of Experimental Biology and approved by the Ethics Committee on Animal Use Experimentation of the Federal University of Pampa, Uruguaiana, Rio Grande do Sul, Brazil (protocol 05/2014). The rats (Male Wistar rats; age 3 mo; 250–300 g) were kept in controlled light, temperature, and humidity conditions (12/12 h light/dark cycle; 23 ± 2°C; 50 ± 10%, respectively) with free access to food and water and randomly submitted to one of the following protocols for 60 days: (I) intramuscular injections (i.m.) of saline solution 0.9% and tap water by gavage – Untreated group; (II) 4.6 μg/kg of HgCl_2_ i.m. for the first dose and subsequent doses of 0.07 μg/kg/day, to cover daily loss, using the model described previously (Rizzetti et al., 2017c) and tap water by gavage – HgCl_2_ group; (III) saline solution 0.9% i.m. and EWH from pepsin for 8 h diluted in tap water (1 g/kg/day), by gavage, according to the model prior reported (Rizzetti et al., 2016a) – EWH group, or (IV) the combination of the HgCl_2_ i.m. and EWH by gavage – EWH + HgCl_2_ group. Bodyweight, food, and water intakes were measured once a week.

This experimental model of chronic controlled exposure to a low concentration of Hg has a total metal concentration in the blood at the end of the treatment of 3.04 ng/ml (Rizzetti et al., 2017c), which is within the safety limit established by US Environmental Protection Agency’s (5.8 ng/ml) and similar to the blood Hg concentration of people exposed to the metal by the workplace or through diet (Rice, 2004). Moreover, to make the model more similar to the human exposure condition, we carry out a simultaneous treatment of Hg and EWH, considering that humans are rarely entirely free of any level of exposure to this metal. So our concern was to investigate the benefits of EWH during continuous and concurrent exposure to Hg.

### Systolic Blood Pressure

Systolic Blood Pressure (SBP) was recorded once a week by tail plethysmography (ADInstruments Pty Ltd., Bella Vista, NSW, Australia) according to Rizzetti et al. (2017c). The animals were preheating the animals at 37°C for 10 min to make the pulsations of the caudal artery detectable, followed by 10 sequential cycles of tail inflation-deflation. The SBP was considered the average of all measures.

### Vascular Experiments in the Mesenteric Arteries

The MRA from anesthetized (ketamine-xylazine, 87 and 13 mg/kg i.p.) rats (diameter of third-order branch, in μm: Untreated: 262 ± 5.1; HgCl_2_: 301 ± 4.8; EWH: 254 ± 7.0 and EWH + HgCl_2_: 318 ± 5.4; *p* > 0.05) were removed and rings of 2 mm in length were mounted in an isometric small-vessel myograph (Multi Wire Myograph System, DMT620, ADInstruments, Australia) according to Mulvany and Halpern (1977) and connected to an acquisition system (PowerLab 8/35, ADInstruments, Australia). Briefly, the rings were submerged in warmed (37°C) Henseleit solution (KHS, in mM at 37°C: 115 NaCl, 25 NaHCO_3_, 4.7 KCl, 1.2 MgSO_4_ 7H_2_O, 2.5 CaCl_2_, 1.2 KH_2_PO_4_, 11.1 glucose, and 0.01 Na_2_EDTA) continuously bubbled with carbogen (5% CO_2_ in O_2_). Initially, segments were contractility contracted with KCl (120 mM) to test the viability of the vessels. The endothelium of some vessels was removed by rubbing the intimal surface. The absence of acetylcholine (ACh – 0.01 nM – 30 mM)-induced relaxation in rings pre-contracted by noradrenaline (NE 10 μM) was taken as an indicator of successful endothelium denudation. Endothelium-independent relaxation were tested by sodium nitroprusside (SNP – 0.1 nM − 3.5 mM) under the same pre-contracted conditions (NE – 10μm). We also evaluated the vascular response to increasing concentrations of NE (10 nM – 30 μM). To evaluate the pathways involved in the contractile responses, the following drugs were added 30 min before the generation of the NE concentration-response curves: nitric oxide synthase (NOS) inhibitor N-nitro-L-arginine methyl ester (L-NAME, 100 mM), a NAD(P)H oxidase inhibitor (VAS2870, 10 μM), an essential cofactor for NO synthesis, tetrahydrobiopterin (BH4, 100 μM), a superoxide dismutase mimetic (TEMPOL, 10 μM), and a non-selective COX inhibitor (Indomethacin, 10 μM).

### Vascular *in situ* Nitric Oxide and Reactive Oxygen Species Detection

Measurements of NO and ROS levels in MRA segments were performed as previously published by Martin et al. (2012) and Avendaño et al. (2014), respectively. Briefly, the MRA rings were incubated for 45 min with 4,5-diaminofluorescein diacetate (DAF-2, 10 μM) to assess the release of NO. The first collection of the medium measured the basal release of NO, and after the segments were incubated with NE 0.1 nM and relaxed with ACh 10 μM, the medium was collected again, and the induced release of NO was measured by fluorescence method (excitation at 492 nm and emission at 515 nm – SpectraMax M5 Molecular Devices, Sunnyvale, CA, United States). The results are expressed as arbitrary units/g tissue as a percentage of fluorescence obtained for Untreated rats. The release of NO is the result of fluorescence evoked by ACh subtracted from the basal release of NO.

For ROS measured, MRA rings were dissected, frozen in OCT solution and then cut in 14-μm-thick sections, placed on a glass slide, and equilibrated in a Krebs-HEPES buffer (in mM: 130 NaCl, 5.6 KCl, 2 CaCl_2_, 0.24 MgCl_2_, and 8.3 HEPES), and 11 glucose, pH 7.4. DHE (2 μM, 30 min, 37°C) incubated for 30 min in a dark-chamber and then viewed by a fluorescence microscope (Eclipse 50i55i Epi-fluorescence Nikon, Tokyo, Japan, magnification: × 40). The total ring area was analyzed using Image J version V1.56 (National Institutes of Health, Bethesda, Maryland, United States) software.

### Biochemical Assays

The supernatant fraction derived from homogenization (50 mM Tris-HCl, pH 7.4, 1/10, w/v) and centrifugation (2,400 *g*; 10 min; 4°C) of the MRA was frozen at −80°C. The levels of reactive species (RS) were measured according to Loetchutinat et al. (2005) by a spectrofluorometric method. Briefly, after diluted (1:5 in Tris-HCl 50 mM; pH 7.4), samples received 1 mM of 2', 7'-dichlorofluorescein diacetate (DCHF-DA). The 2', 7'-dichlorofluorescein (DCF) fluorescence intensity emission represents the amount of ROS formed (520 nm emission with 480 nm excitation for 60 min at 15 min intervals – SpectraMax M5 Molecular Devices, CA, United States). The ROS levels were expressed as fluorescence units and as a percentage of those obtained for Untreated rats.

The malondialdehyde (MDA) levels that represent the lipid peroxidation were measured following Ohkawa et al. (1979) protocol with modifications by the colorimetric method. Briefly, thiobarbituric acid (TBA) 0.8%, acetic acid buffer (pH 3.2) plus sodiumduodecilsulphate (SDS) 8% were added to the samples and the color reaction was measured against blanks (532 nm – SpectraMax M5 Molecular Devices, Sunnyvale, CA, United States). The data were expressed as nmol of MDA per gram of tissue.

The total antioxidant capacity was measured by Ferric Reducing/Antioxidant Power (FRAP) assay, according to Benzie and Strain, 1996. Briefly, homogenate of MRA was added to FRAP reagent [acetate buffer pH 3.6, 10 mM; 2,4,6-Tri(2-piridil)-s-triazina – TPTZ, 40 mM HCl; FeCl3-10:1:1; 37°C] and incubated at 37°C for 10 min. The absorbance was read at 593 nm (SpectraMax M5 Molecular Devices, CA, United States). The standard dose-response curve of Trolox (50–1,000 μM) was performed, and results are presented with particular reference to Trolox equivalents.

Non-protein thiols (NPSH) were measured, according to Ellman (1959). MRA tissue was added to a buffer of potassium phosphate (1 M, pH 7.4), and 5,5'-dithio-bis(2-nitrobenzoic acid) (DTNB, 10 mM) and the color reaction was spectrophotometrically read (412 nm – SpectraMax M5 Molecular Devices, CA, United States). The results were expressed as nmol of NPSH per gram of tissue.

### Statistical Analyses

Values are expressed as means ± SEM. The results were analyzed using a two-way ANOVA. When two-way ANOVA showed a statistical significance, the Fisher *post hoc* test was applied (Graph Pad Prism 6.0 Software, San Diego, CA). To compare the effects between groups (endothelium removal and drugs) on the response to NE, some results are expressed as the differences of areas under the concentration-response curves (dAUC) in the control and experimental situations. The differences were expressed as the % of the AUC of the corresponding control situation. The results were considered statistically significant for values of *p* < 0.05.

## Results

The water and food intake (data not shown) as well body weight gain were not modified during the treatment (body weight gain, in g: Untreated: 58.5 ± 4.1; HgCl_2_: 60.3 ± 4.9; EWH: 59.7 ± 3.8; EWH + HgCl_2_: 61.1 ± 5.3; *n* = 8; and *p* > 0.05). Increased SBP values were observed in HgCl_2_-treated rats and EWH treatment was able to decrease SBP values in HgCl_2_-treated rats (SBP values, in mmHg: Untreated: 120.1 ± 1.9; HgCl_2_: 135.2 ± 2.8^*^; EWH: 124.5 ± 1.5; EWH + HgCl_2_: 122.0 ± 2.2^#^; *n* = 8; and *p* < 0.05 – ^*^ vs. Untreated and ^#^ vs. HgCl_2_). In MRA reactivity, the maximum response to KCl was similar between groups (in mN/mm, Untreated: 14.1 ± 0.3; HgCl_2_: 14.2 ± 0.5; EWH: 14.3 ± 0.1; EWH + HgCl_2_: 14.1 ± 0.2; *n* = 8; and *p* > 0.05), demonstrating that neither Hg nor EWH alter vascular integrity of MRA.

**Figure 1:**
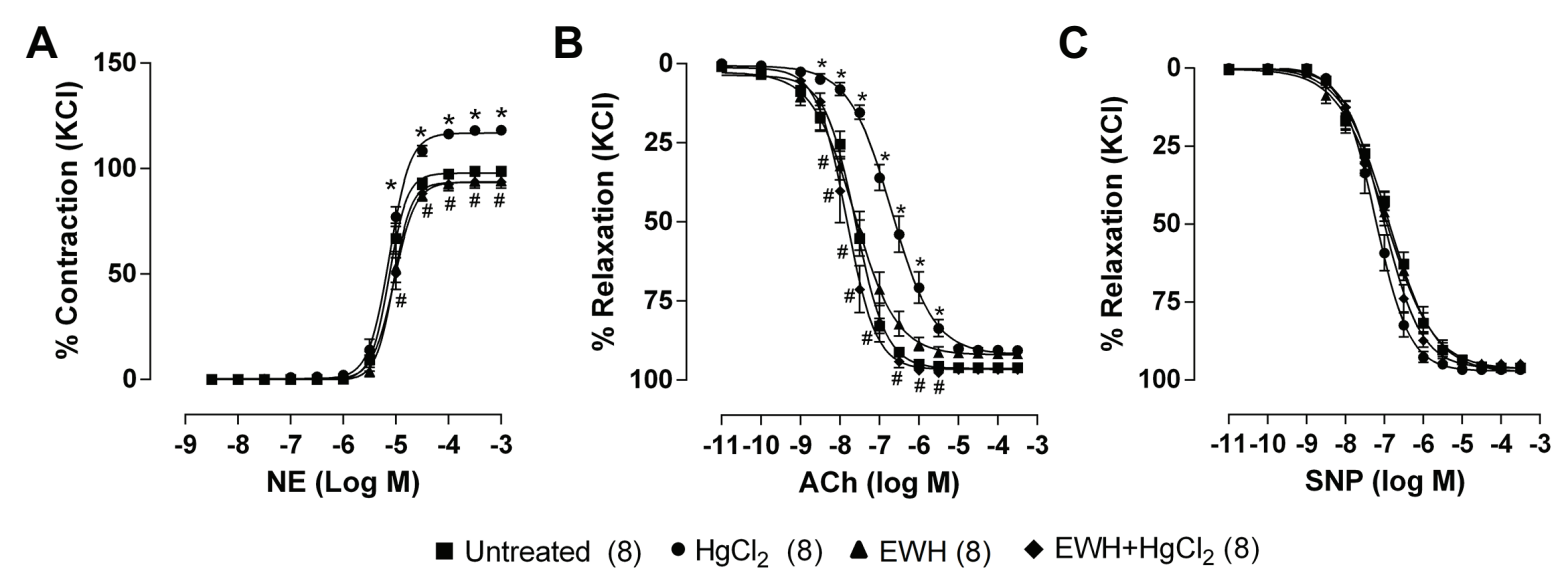
Effect of egg white hydrolysate (EWH) co-treatment in mesenteric resistance arteries (MRA) reactivity of rats exposure to mercury chloride (HgCl_2_) for 60 days. Concentration–response curves to **(A)** noradrenaline (NE), **(B)** acetylcholine (ACh), and **(C)** sodium nitroprusside (SNP) in MRA segments. The results (mean ± SEM) are expressed as a percentage of the response to 120 mmol/l KCl. Two-Way ANOVA followed by Fisher test; n of each group in parenthesis; ^*^p < 0.05 vs. Untreated and ^#^ vs. HgCl_2_.

Egg white hydrolysate intake prevented the increased contractile responses to NE and the reduced endothelium-dependent vasodilator response to ACh induced by HgCl_2_ exposure. The endothelium-independent vasodilator response to NPS in MRA was not affected in all treatments (Figures 1A–C).

**Figure 2:**
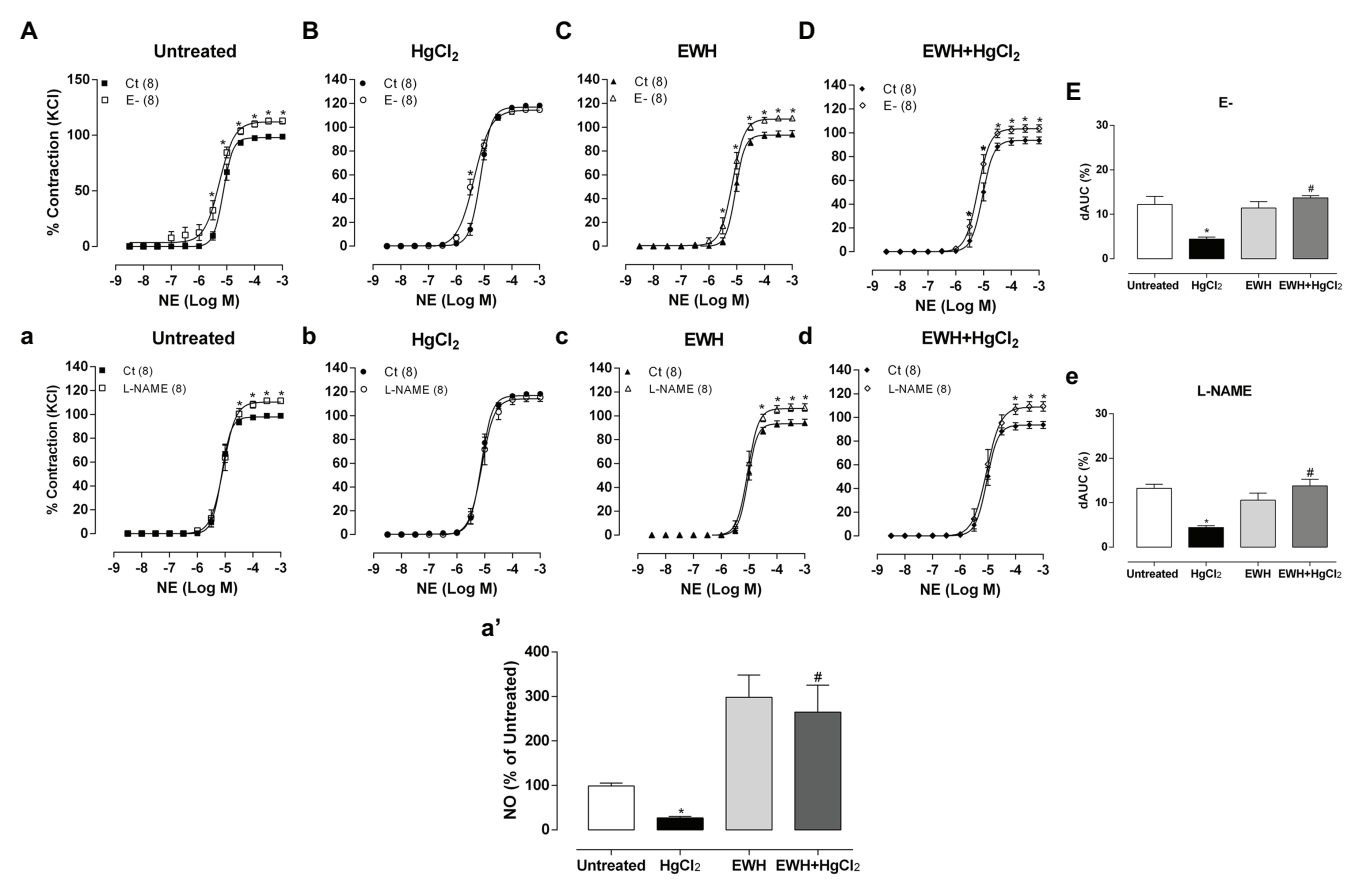
Effects of EWH co-treatment in MRA of rats exposure to HgCl_2_ for 60 days on the endothelium and nitric oxide (NO) mediated vascular response. Concentration-response curve to NE in the absence of endothelium (E−; **A−D**) and N-nitro-L-arginine methyl ester (L-NAME; 100 μM; **a–d**) in mesenteric segments from rats Untreated **(A)**, treated with HgCl_2_ **(B)**, with EWH **(C)**, and with EWH + HgCl_2_ **(D)**. The results (mean ± SEM) are expressed as a percentage of the response to 120 mmol/l KCl. Two-Way ANOVA followed by Fisher test; ^*^p < 0.05 vs. Untreated and ^#^ vs. HgCl_2_. Differences in the area under the concentration-response curves (dAUC) in mesenteric segments are represented in (**E**,**e**) of the four experimental groups; one-way ANOVA, ^*^p < 0.05 vs. Untreated and ^#^ vs. HgCl_2_. In **(a’)** represent the NO release in all experimental groups.

Denuded endothelium or NOS inhibitor incubation (L-NAME) increased the contractile response to NE in all groups except in HgCl_2_ group as evidenced by the dAUC values (Figures 2A–E,a–e). These findings show the absence of endothelial participation in the vasoconstrictor response to NE in this group. MRA segments from HgCl_2_ exposure animals that received EWH showed similar effects of endothelium removal or L-NAME compared to the Untreated group (Figures 2A–E,a–e), suggesting that EWH prevented this reduced endothelial modulation by NO. In agreement, ACh-induced NO release was lower in MRA from Hg-treated rats. On the other hand, rats receiving EWH alone and those receiving the combination of HgCl_2_ and EWH had a greater percentage of NO release when compared to the Untreated group (Figure 2a’). This finding suggests that EWH was able to induce NO production by NOS.

**Figure 3:**
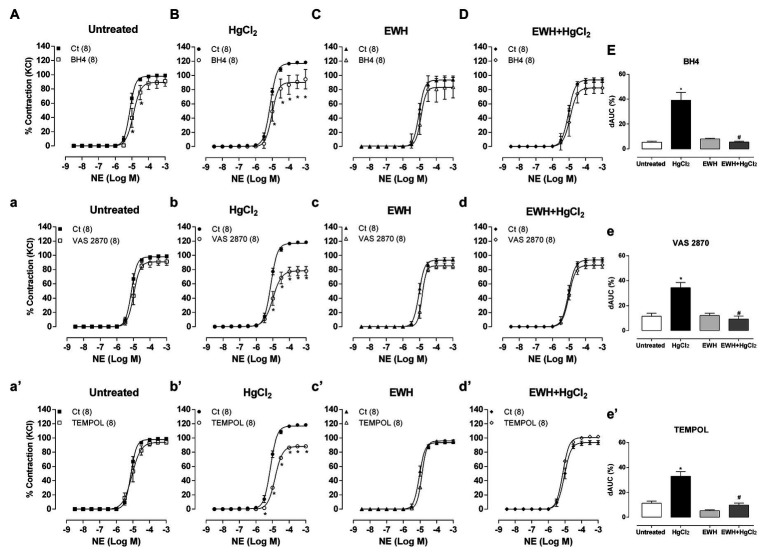
Effects of EWH on NO cofactor modulation, participation of reactive oxygen species (ROS) from NAD(P)H oxidase and participation of superoxide anion in vasoconstrictor responses to NE in MRA from rats exposed to low concentrations of HgCl_2_ for 60 days. Concentration-response curve to NE in the absence (Ct), presence of the endothelial nitric oxide synthase (eNOS) cofactor (BH4; **A–D**), the presence of the NAD(P)H synthase inhibitor (VAS2870; **a–d**) and the presence of the superoxide anion scavenger mimetic (TEMPOL; **a’–d’**) in mesenteric segments from rats Untreated **(A)**, treated with HgCl_2_ **(B)**, with EWH **(C)**, and with EWH + HgCl_2_ **(D)**. The results (mean ± SEM) are expressed as a percentage of the response to 120 mmol/l KCl. Differences in the area under the concentration-response curves (dAUC) in mesenteric segments in the presence and the absence of BH4, VAS2870 and TEMPOL of the four experimental groups (**E**,**e**,**e’**); n of each group in parenthesis, one-way ANOVA, ^*^p < 0.05 vs. Untreated and ^#^ vs. HgCl_2_.

The cofactor for NO synthesis BH4 incubation had a smaller contractile response to NE in HgCl_2_-treated rats, showing lower BH4 bioavailability and, possibly, an uncoupled state of endothelial nitric oxide synthase (eNOS) in this vessel. EWH intake maintained BH4 bioavailability in MRA from HgCl_2_-treated rats, indicating the improvement of eNOS and NO synthesis (Figures 3A–E).

**Figure 4:**
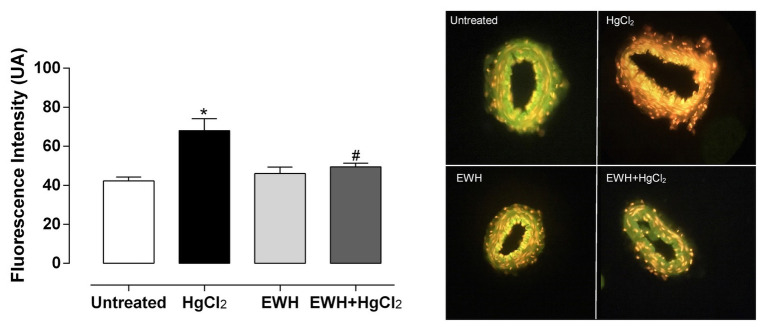
Effects of EWH on local anion superoxide production in mesenteric of rats exposed to low concentrations of HgCl_2_ for 60 days. Superoxide Anion production in mesenteric from rats Untreated, treated HgCl_2_, with EWH and with EWH + HgCl_2_ levels in mesenteric of all groups; n of each group in parenthesis, one-way ANOVA, ^*^p < 0.05 vs. Untreated, ^#^ vs. HgCl_2_.

**Figure 5:**
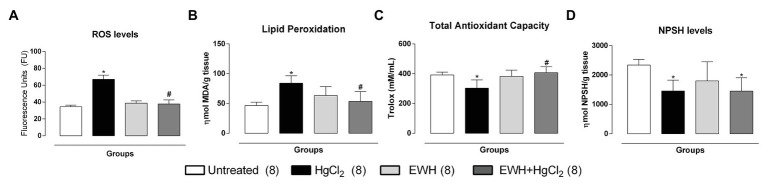
Effects of EWH in the ROS **(A)**, MDA **(B)**, the total antioxidant capacity **(C)**, and NPSH **(D)** in mesenteric of rats exposed to low concentrations of HgCl_2_ for 60 days. Data are expressed as mean ± SEM. Number of animals is indicated in parentheses respectively, one-way ANOVA, ^*^p < 0.05 vs. Untreated; ^#^ vs. HgCl_2_.

The NAD(P)H oxidase inhibitor VAS2870 reduced the contractile response to NE in MRA only in HgCl_2_ group (Figures 3a–e). EWH prevented the increased ROS participation from NAD(P)H oxidase on contractile response to NE. The superoxide dismutase mimetic TEMPOL reduced the contractile response to NE in MRA only from HgCl_2_-exposed rats; thus, the NE responses remained unchanged in the other groups (Figures 3a’–e’). We also observed significantly higher superoxide anion production in arteries of rats exposed to HgCl_2_; EWH prevented this effect (Figure 4). Chronic Hg treatment for 60 days increased ROS levels and lipid peroxidation in MRA from exposed rats (Figures 5A,B) and reduced the total antioxidant capacity and the NPHS levels in the Hg-exposed rats (Figures 5C,D). EWH was able to prevent the oxidative stress in MRA of HgCl_2_-treated rats, balancing the pro-oxidant and antioxidant status (Figures 5A–D).

**Figure 6:**
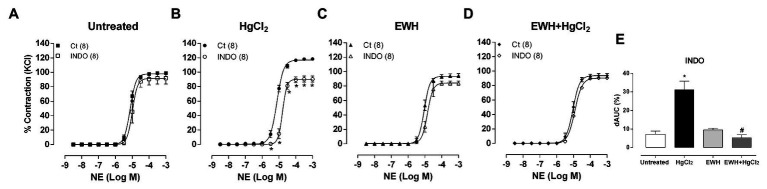
Effects of EWH on contribution of cyclooxygenase (COX) pathway on vasoconstrictor responses to NE in mesenteric of rats exposed to low concentrations of HgCl_2_ for 60 days. Concentration-response curve to NE in the absence (Ct), the presence of the non-selective COX inhibitor (Indomethacin; **A–D**) in mesenteric segments from rats Untreated **(A)**, treated with HgCl_2_ **(B)**, with EWH **(C)**, and with EWH + HgCl_2_ **(D)**. The results (mean ± SEM) are expressed as a percentage of the response to 120 mmol/l KCl. Two-Way ANOVA followed by Fisher test; n of each group in parenthesis; ^*^p < 0.05 vs. Untreated and ^#^ vs. HgCl_2_. Differences in the area under the concentration-response curves (dAUC) in mesenteric segments are represented in **(E)** graph (one-way ANOVA).

Indomethacin reduced the response to NE in MRA segments from only in HgCl_2_-treated rats (Figures 6A–E) indicating that the enhanced COX pathway participation in these responses was prevented by EWH.

## Discussion

Intake of EWH as a functional food was able to reverse the increase in SBP induced by chronic exposure to HgCl_2_ at low concentrations, which is related to the reduction of contractile responses and the vascular dysfunction induced by the metal in MRA. These effects were associated, at least in part, with the capacity of EWH to produce NO from eNOS and with its antioxidant and anti-inflammatory properties. The bioactive peptides of EWH protect against high concentrations of ROS from NAD(P)H oxidase and, possibly, activation of inflammatory COX in MRA in HgCl_2_-treated rats, thus normalizing the NO modulation in the vasculature.

The Hg is a well-known environmental risk factor for cardiovascular diseases (Virtanen et al., 2007). Acute Hg exposure promotes the reduction of myocardial contractility and inhibition of myosin ATPase activity (Vassallo et al., 1999). Moreover, subchronic exposure to Hg, at doses similar to human exposure, increases vascular reactivity of resistance and conductance vessels in rats (Wiggers et al., 2008; Peçanha et al., 2010). A prolonged exposure at low doses of HgCl_2_ for 60 days increased SBP and vascular dysfunction in conductance arteries; these effects were related to stimulation of ACE activity, NAD(P)H oxidase-mediated oxidative stress and activation of COX-2 inflammatory pathway in these vessels (Rizzetti et al., 2017a,c). Our results demonstrate that resistance arteries are also affected by prolonged exposure to Hg, which could better explain the high SBP values observed, since the main vascular site responsible for vascular resistance and maintenance of BP is the resistance arteries (Oparil et al., 2018). Our purpose was to investigate if the mechanisms involved in this increment of SBP could be blocked by EWH treatment.

The Hg exerts its toxic effects on the cardiovascular system through oxidative stress caused by the production of superoxide anion from NAD(P)H oxidase and, possibly, inflammatory mediators derived from COX. Although we have not verified the specific participation of the COX-2 pathway in the current study, previous work of our group, at the same model, evidenced the participation of this pathway in increasing vascular reactivity in conductance arteries using a selective COX-2 inhibitor (NS 398), and the relationship between ROS and COX-2-derived prostanoids. Moreover, the vascular functional findings showed a reduction in the COX-2 participation in the cardiovascular system of Hg-treated rats after EWH intake, proving its anti-inflammatory property observed *in vitro* (Rizzetti et al., 2017a). Thus, in this study, we suggest that inflammatory mediators derived from COX-2 are also involved in increasing vascular reactivity in resistance arteries. However, future investigations using selective inhibitors of this pathway may explore these findings.

Besides, we verified for the first time the possible involvement of eNOS uncoupling in HgCl_2_-induced negative actions on vascular tissue; this could be due to high vascular oxidative stress caused by inflammatory stimuli from the COX pathway. Our findings demonstrated the protective effect of EWH on resistance arteries, which blocked the mechanisms involved in the increment of blood pressure by Hg.

Egg white hydrolysate derived from hydrolysis with pepsin for 8 h has several biological activities, such as antioxidant, free radical scavenger, ACE inhibitor, vascular-relaxing, and anti-inflammatory (Miguel et al., 2004). Fourteen of its constituent peptides have been identified (FRADHPFL, RADHPFL, YAEERYPIL, YRGGLEPINF, ESIINF, RDILNQ, IVF, YQIGL, SALAM, and FSL; Davalos et al., 2004; Miguel et al., 2004; Garcés-Rimón et al., 2016) and their biological actions have been previously reported *in vitro* or *in vivo* studies (Miguel et al., 2006, 2007; Garces-Rimon et al., 2019). However, these peptides and amino acids act individually, cooperatively, and synergistically; during their passage through the gastrointestinal tract, some modifications due to new hydrolysis should be considered (Miner-Williams et al., 2014). Thus, we do not know if the effects observed in this study are due to a specific peptide or the sum of all its constituent peptides’ effects. Although most studies conducted in recent years have focused on the isolation of peptide sequences released during hydrolysis, it has recently been proven that the administration of complete hydrolysates could be more relevant than the administration of a single isolated peptide since a more significant biological effect could be achieved (Liu et al., 2017). Moreover, we consider that hydrolysates could be more attractive products for developing functional foods from a technological and organoleptic point of view.

In the present study, we demonstrated improvements in resistance arteries function and, consequently, in SBP levels of Hg-treated rats after the EWH treatment. The mechanisms involved on cardiovascular beneficial effects observed after consumption of EWH are probably due to its vascular-relaxing, antioxidant and anti-inflammatory effects. The antihypertensive capacity of EWH we show here was previously reported in experimental models of spontaneously hypertensive rats (SHR) (Miguel et al., 2006) and was attributed, at least in part, to the vasodilator peptides whose N-terminal position exhibits amino acids Arg or Tyr (Miguel et al., 2004, 2005).

It has been previously reported that alterations in eNOS gene increase the susceptibility to cardiovascular diseases in individuals after Hg exposure by modulating NO levels (de Marco et al., 2012). Exposure to Hg at low concentrations has deleterious vascular effects on aorta, coronary and basilar arteries related to greater vascular reactivity caused by the reduction of NO bioavailability in these vessels (Wiggers et al., 2008; Peçanha et al., 2010; Botelho et al., 2019). Moreover, a study in mesenteric arteries from rats exposed for 30 days to HgCl_2_ showed eNOS protein expression upregulation possibly due to a compensatory mechanism against metal-induced endothelial dysfunction (Wiggers et al., 2008). Our results indicate that lower NO bioavailability and endothelial dysfunction persists in MRA of long-term Hg exposure. Moreover, we showed that lower NO bioavailability occurs, at least in part, due to less NO release in resistance arteries.

Considering previous and present findings, we could hypothesize that the increased eNOS protein expression in MRA can accompany the reduction of this isoform activity or that its expression and activity may be increased. However, this enzyme could be decoupled and producing ROS, which would support the increase in oxidative stress associated with reducing the NO release observed in this study. In this condition, eNOS shifts from NO production to overproducing superoxide anion, increasing oxidative stress in the vasculature (Antoniades et al., 2006). Here we observed lower BH4 bioavailability in MRA of Hg-treated rats, which represents an important cofactor for the production of NO by this enzyme. This finding suggests that Hg induces eNOS uncoupling by inhibiting its cofactor. In any case, the alteration of eNOS protein expression induced by exposure to Hg increases, oxidative stress, and vascular damage are being protected by the ingestion of EWH.

In this sense, NO bioavailability could also be related to oxidative stress imbalance due to overproduction of vascular ROS, generation of peroxynitrite, and reduction of antioxidant reserves (Faria et al., 2018). Interestingly, oxidative stress has also been reported as an important mechanism responsible for lower NO bioavailability induced by Hg (Wiggers et al., 2008; Rizzetti et al., 2017c); it is also involved in Hg-induced vascular damage in our study. Besides, oxidative stress can directly modify eNOS protein or its cofactor BH4, leading to enzymatic dysfunction in vascular tissue (Dumitrescu et al., 2007).

It is important to emphasize that EWH is composed of several peptides that have vasodilator effects (Miguel et al., 2007). These peptides were related to EWH capacity for increasing NO release by the rise in eNOS activity or the upregulation in protein expression of endothelial cells (Miguel et al., 2020). Our study suggests that EWH was able to increase the NO release possibly by enhancing the eNOS function, which is the major isoform involved in the control of vascular function and blood pressure. The vasodilator power of EWH in SHR models is related to Arg or Tyr peptide content necessary in the N-terminal position for vasodilator activity (Garcia-Redondo et al., 2010).

An alternative mechanism implied on the vasodilator activity of the EWH and derived peptides is its action mediated by NO production *via* the bradykinin B1 receptor (Miguel et al., 2007). Others authors showed that egg ovotransferrin-derived peptides increased NO-mediated vasodilation in MRA of SHR animals through increasing eNOS expression (Majumder et al., 2013). Future studies are required to elucidate the mechanisms of action by which EWH acts on the expression and activity of eNOS.

In the present study, EWH was able to prevent oxidative stress in MRA of Hg-treated rats, avoiding superoxide anion generation from NAD(P)H oxidase and balancing pro-oxidant and antioxidant status verified by the functional experiments and the oxidative stress biomarkers in vascular tissue. Long-term Hg exposed-rats had lower NOX-4 and p22phox mRNA levels in aorta after co-treatment with EWH; this was related to lower vascular reactivity in conductance vessels (Rizzetti et al., 2017a). A similar effect could be found in MRA in the present study to explain our findings. Previous studies showed that antioxidant EWH properties decreased plasmatic MDA levels and increased the levels of low glutathione in the liver of Zucker obese animals (Garcés-Rimón et al., 2016). Restored plasma antioxidant capacity was found in high-fat/high-dextrose fed rats with metabolic syndrome after EWH intake (Moreno-Fernandez et al., 2018a). Also, normalized oxidative biomarkers were reported in neurological, reproductive and cardiovascular systems from Hg-exposed animals after EWH consumption (Rizzetti et al., 2016a,b, 2017a,b).

In summary, EWH intake promotes protective effects against the endothelial dysfunction in MRA, and consequently, the increase in SBP of long-term HgCl_2_-exposed rats. EWH benefits could be related to its NO-induced vasodilatation capacity and its antioxidant and anti-inflammatory properties. EWH reduces high ROS generation by NAD(P)H oxidase and, possibly, the activation of inflammatory COX in MRA from Hg-treated rats. Our findings strongly suggest that dietary supplementation with EWH may represent an important strategy for counteracting the effects of cardiotoxicity by pro-oxidant agents such as heavy metals.

## Data Availability Statement

The original contributions presented in the study are included in the article/Supplementary Material, further inquiries can be directed to the corresponding author.

## Ethics Statement

The animal study was reviewed and approved by Ethics Committee on Animal Use Experimentation of the Federal University of Pampa, Uruguaiana, Rio Grande do Sul, Brazil (protocol 05/2014).

## Author Contributions

DR, FP, DV, MM, and GW conceived and designed the experiments. AE, DR, and JP performed the experiments. AE, DR, FP, and GW analyzed the data. DV, MM, and GW contributed reagents, materials and analysis tools. AE, DR, DV, MM, and GW wrote the paper. All authors contributed to the article and approved the submitted version.

### Conflict of Interest

The authors declare that the research was conducted in the absence of any commercial or financial relationships that could be construed as a potential conflict of interest.

## Abbreviations

ACE: Angiotensin-converting enzyme
ACh: Acetylcholine
BH4: Tetrahydrobiopterin
COX: Cyclooxygenase
DAF: 2-4,5-diaminofluorescein diacetate
dAUC: Areas under the concentration-response curves
DCF: 2', 7'-dichlorofluorescein
DCHF-DA: 2', 7'-dichlorofluorescein diacetate
DTNB: 5,5'-dithio-bis(2-nitrobenzoic acid)
EWH: Egg white hydrolysate
Fe2+: Ferrous ion
Fe3+: Ferric ion
FRAP: Ferric Reducing/Antioxidant Power
Hg: Mercury
HgCl2: Mercury chloride
i.m.: Intramuscular injections
L-NAME: N-nitro-L-arginine methyl ester
MDA: Malondialdehyde
MRA: Resistance mesenteric arteries
NE: Noradrenaline
NO: Nitric oxide
NOS: Nitric oxide synthase
NPSH: Non-protein thiols
ROS: Reactive oxygen species
RS: Reactive species
SBP: Systolic blood pressure
SDS: Sodiumdodecilsulphate
SHR: Spontaneously hypertensive rats
SNP: Sodium nitroprusside
TBA: Thiobarbituric acid
TPTZ: 2,4,6-Tri(2-piridil)-s-triazina

## References

[ref1] Antoniades C. Shirodaria C. Warrick N. Cai S. de Bono J. Lee J.. (2006). 5-methyltetrahydrofolate rapidly improves endothelial function and decreases superoxide production in human vessels: effects on vascular tetrahydrobiopterin availability and endothelial nitric oxide synthase coupling. Circulation 114, 1193–1201. 10.1161/CIRCULATIONAHA.106.612325, PMID: 16940192

[ref2] Avendaño M. S. Lucas E. Jurado-Pueyo M. Martinez-Revelles S. Vila-Bedmar R. Mayor F. Jr.. (2014). Increased nitric oxide bioavailability in adult GRK2 hemizygous mice protects against angiotensin II-induced hypertension. Hypertension 63, 369–375. 10.1161/HYPERTENSIONAHA.113.01991, PMID: 24191280

[ref3] Benzie I. F. Strain J. J. (1996). The ferric reducing ability of plasma (FRAP) as a measure of “antioxidant power”: the FRAP assay. Anal. Biochem. 239, 70–76. 10.1006/abio.1996.0292, PMID: 8660627

[ref4] Botelho T. Marques V. B. Simoes M. R. do Val Lima P. R. Simoes F. V. Vassallo D. V.. (2019). Impaired participation of potassium channels and Na(+)/K(+) -ATPase in vasodilatation due to reduced nitric oxide bioavailability in rats exposed to mercury. Basic Clin. Pharmacol. Toxicol. 124, 190–198. 10.1111/bcpt.13113, PMID: 30125472

[ref5] Chikara S. Nagaprashantha L. D. Singhal J. Horne D. Awasthi S. Singhal S. S. (2018). Oxidative stress and dietary phytochemicals: role in cancer chemoprevention and treatment. Cancer Lett. 413, 122–134. 10.1016/j.canlet.2017.11.002, PMID: 29113871

[ref6] Davalos A. Miguel M. Bartolome B. Lopez-Fandino R. (2004). Antioxidant activity of peptides derived from egg white proteins by enzymatic hydrolysis. J. Food Prot. 67, 1939–1944. 10.4315/0362-028X-67.9.1939, PMID: 15453585

[ref7] de Marco K. C. Antunes L. M. Tanus-Santos J. E. Barbosa F. Jr. (2012). Intron 4 polymorphism of the endothelial nitric oxide synthase (eNOS) gene is associated with decreased NO production in a mercury-exposed population. Sci. Total Environ. 414, 708–712. 10.1016/j.scitotenv.2011.11.010, PMID: 22134029

[ref8] Dumitrescu C. Biondi R. Xia Y. Cardounel A. J. Druhan L. J. Ambrosio G.. (2007). Myocardial ischemia results in tetrahydrobiopterin (BH4) oxidation with impaired endothelial function ameliorated by BH4. Proc. Natl. Acad. Sci. U. S. A. 104, 15081–15086. 10.1073/pnas.0702986104, PMID: 17848522 PMC1986616

[ref9] Ellman G. L. (1959). Tissue sulfhydryl groups. Arch. Biochem. Biophys. 82, 70–77. 10.1016/0003-9861(59)90090-6, PMID: 13650640

[ref10] Faria T. O. Simoes M. R. Vassallo D. V. Forechi L. Almenara C. C. P. Marchezini B. A.. (2018). Xanthine oxidase activation modulates the endothelial (vascular) dysfunction related to HgCl2 exposure plus myocardial infarction in rats. Cardiovasc. Toxicol. 18, 161–174. 10.1007/s12012-017-9427-x, PMID: 28980197

[ref11] Garces-Rimon M. Gonzalez C. Hernanz R. Herradon E. Martin A. Palacios R.. (2019). Egg white hydrolysates improve vascular damage in obese zucker rats by its antioxidant properties. J. Food Biochem. 43:e13062. 10.1111/jfbc.13062, PMID: 31571257

[ref12] Garcés-Rimón M. Gonzalez C. Uranga J. A. Lopez-Miranda V. Lopez-Fandino R. Miguel M. (2016). Pepsin egg white hydrolysate ameliorates obesity-related oxidative stress, inflammation and steatosis in zucker fatty rats. PLoS One 11:e0151193. 10.1371/journal.pone.0151193, PMID: 26985993 PMC4795625

[ref13] Garcia-Redondo A. B. Roque F. R. Miguel M. Lopez-Fandino R. Salaices M. (2010). Vascular effects of egg white-derived peptides in resistance arteries from rats. Structure-activity relationships. J. Sci. Food Agric. 90, 1988–1993. 10.1002/jsfa.4037, PMID: 20572060

[ref14] Genchi G. Sinicropi M. S. Carocci A. Lauria G. Catalano A. (2017). Mercury exposure and heart diseases. Int. J. Environ. Res. Public Health 14:74. 10.3390/ijerph14010074, PMID: 28085104 PMC5295325

[ref15] Halliwell B. (2007). Oxidative stress and cancer: have we moved forward? Biochem. J. 401, 1–11. 10.1042/BJ20061131, PMID: 17150040

[ref16] Incalza M. A. D’Oria R. Natalicchio A. Perrini S. Laviola L. Giorgino F. (2018). Oxidative stress and reactive oxygen species in endothelial dysfunction associated with cardiovascular and metabolic diseases. Vascul. Pharmacol. 100, 1–19. 10.1016/j.vph.2017.05.005, PMID: 28579545

[ref17] Liguori I. Russo G. Curcio F. Bulli G. Aran L. Della-Morte D.. (2018). Oxidative stress, aging, and diseases. Clin. Interv. Aging 13, 757–772. 10.2147/CIA.S158513, PMID: 29731617 PMC5927356

[ref18] Liu F. Ma C. Gao Y. McClements D. J. (2017). Food grade covalent complexes and their application as nutraceutical delivery systems: a review. Compr. Rev. Food Sci. F. 16, 76–95. 10.1111/1541-4337.12229 33371544

[ref19] Loetchutinat C. Kothan S. Dechsupa S. Meesungnoen J. Jay-gerin J. P. Mankhetkorn S. (2005). Spectrofluorometric determination of intracellular levels of reactive oxygen species in drug-sensitive and drug-resistant cancer cells using the 2',7'-dichlorofluorescein diacetate assay. Radiat. Phys. Chem. 72, 323–331. 10.1016/j.radphyschem.2004.06.011

[ref20] Majumder K. Chakrabarti S. Morton J. S. Panahi S. Kaufman S. Davidge S. T.. (2013). Egg-derived tri-peptide irw exerts antihypertensive effects in spontaneously hypertensive rats. PLoS One 8:e82829. 10.1371/journal.pone.0082829, PMID: 24312436 PMC3843735

[ref21] Manso M. A. Miguel M. Even J. Hernandez R. Aleixandre A. Lopez-Fandino R. (2008). Effect of the long-term intake of an egg white hydrolysate on the oxidative status and blood lipid profile of spontaneously hypertensive rats. Food Chem. 109, 361–367. 10.1016/j.foodchem.2007.12.049, PMID: 26003359

[ref22] Martin A. Perez-Giron J. V. Hernanz R. Palacios R. Briones A. M. Fortuno A.. (2012). Peroxisome proliferator-activated receptor-gamma activation reduces cyclooxygenase-2 expression in vascular smooth muscle cells from hypertensive rats by interfering with oxidative stress. J. Hhypertens. 30, 315–326. 10.1097/HJH.0b013e32834f043b, PMID: 22179086

[ref23] Miguel M. Lopez-Fandino R. Ramos M. Aleixandre A. (2005). Short-term effect of egg-white hydrolysate products on the arterial blood pressure of hypertensive rats. Br. J. Nutr. 94, 731–737. 10.1079/BJN20051570, PMID: 16277776

[ref24] Miguel M. Lopez-Fandino R. Ramos M. Aleixandre A. (2006). Long-term intake of egg white hydrolysate attenuates the development of hypertension in spontaneously hypertensive rats. Life Sci. 78, 2960–2966. 10.1016/j.lfs.2005.11.025, PMID: 16386762

[ref25] Miguel M. Manso M. Aleixandre A. Alonso M. J. Salaices M. Lopez-Fandino R. (2007). Vascular effects, angiotensin I-converting enzyme (ACE)-inhibitory activity, and antihypertensive properties of peptides derived from egg white. J. Agric. Food Chem. 55, 10615–10621. 10.1021/jf072307o, PMID: 18047278

[ref26] Miguel M. Recio I. Gomez-Ruiz J. A. Ramos M. Lopez-Fandino R. (2004). Angiotensin I-converting enzyme inhibitory activity of peptides derived from egg white proteins by enzymatic hydrolysis. J. Food Prot. 67, 1914–1920. 10.4315/0362-028X-67.9.1914, PMID: 15453581

[ref27] Miguel M. Vassallo D. V. Wiggers G. A. (2020). Bioactive peptides and hydrolysates from egg proteins as a new tool for protection against cardiovascular problems. Curr. Pharm. Des. 26, 3676–3683. 10.2174/1381612826666200327181458, PMID: 32216734

[ref28] Miner-Williams W. M. Stevens B. R. Moughan P. J. (2014). Are intact peptides absorbed from the healthy gut in the adult human? Nutr. Res. Rev. 27, 308–329. 10.1017/S0954422414000225, PMID: 25623084

[ref29] Montezano A. C. Dulak-Lis M. Tsiropoulou S. Harvey A. Briones A. M. Touyz R. M. (2015). Oxidative stress and human hypertension: vascular mechanisms, biomarkers, and novel therapies. Can. J. Cardiol. 31, 631–641. 10.1016/j.cjca.2015.02.008, PMID: 25936489

[ref30] Montezano A. C. Touyz R. M. (2014). Reactive oxygen species, vascular Noxs, and hypertension: focus on translational and clinical research. Antioxid. Redox Signal. 20, 164–182. 10.1089/ars.2013.5302, PMID: 23600794 PMC3880913

[ref31] Moreno-Fernandez S. Garces-Rimon M. Gonzalez C. Uranga J. A. Lopez-Miranda V. Vera G.. (2018a). Pepsin egg white hydrolysate ameliorates metabolic syndrome in high-fat/high-dextrose fed rats. Food Funct. 9, 78–86. 10.1039/c7fo01280b, PMID: 29114652

[ref32] Moreno-Fernandez S. Garcés-Rimón M. Uranga J. A. Astier J. Landrier J. F. Miguel M. (2018b). Expression enhancement in brown adipose tissue of genes related to thermogenesis and mitochondrial dynamics after administration of pepsin egg white hydrolysate. Food Funct. 9, 6599–6607. 10.1039/c8fo01754a, PMID: 30489585

[ref33] Mulvany M. J. Halpern W. (1977). Contractile properties of small arterial resistance vessels in spontaneously hypertensive and normotensive rats. Circ. Res. 41, 19–26. 10.1161/01.res.41.1.19, PMID: 862138

[ref34] Ohkawa H. Ohishi N. Yagi K. (1979). Assay for lipid peroxides in animal tissues by thiobarbituric acid reaction. Anal. Biochem. 95, 351–358. 10.1016/0003-2697(79)90738-3, PMID: 36810

[ref35] Oparil S. Acelajado M. C. Bakris G. L. Berlowitz D. R. Cifkova R. Dominiczak A. F.. (2018). Hypertension. Nat. Rev. Dis. Primers 4:18014. 10.1038/nrdp.2018.14, PMID: 29565029 PMC6477925

[ref36] Peçanha F. M. Wiggers G. A. Briones A. M. Perez-Giron J. V. Miguel M. Garcia-Redondo A. B.. (2010). The role of cyclooxygenase (COX)-2 derived prostanoids on vasoconstrictor responses to phenylephrine is increased by exposure to low mercury concentration. J. Physiol. Pharmacol. 61, 29–36. PMID: 20228412

[ref37] Peluso I. Serafini M. (2017). Antioxidants from black and green tea: from dietary modulation of oxidative stress to pharmacological mechanisms. Br. J. Pharmacol. 174, 1195–1208. 10.1111/bph.13649, PMID: 27747873 PMC5429329

[ref38] Rice D. C. (2004). The US EPA reference dose for methylmercury: sources of uncertainty. Environ. Res. 95, 406–413. 10.1016/j.envres.2003.08.013, PMID: 15220074

[ref39] Rizzetti D. A. Altermann C. D. Martinez C. S. Pecanha F. M. Vassallo D. V. Uranga-Ocio J. A.. (2016a). Ameliorative effects of egg white hydrolysate on recognition memory impairments associated with chronic exposure to low mercury concentration. Neurochem. Int. 101, 30–37. 10.1016/j.neuint.2016.10.002, PMID: 27732885

[ref40] Rizzetti D. A. Fernandez F. Moreno S. Uranga Ocio J. A. Pecanha F. M. Vera G.. (2016b). Egg white hydrolysate promotes neuroprotection for neuropathic disorders induced by chronic exposure to low concentrations of mercury. Brain Res. 1646, 482–489. 10.1016/j.brainres.2016.06.037, PMID: 27350078

[ref41] Rizzetti D. A. Martin A. Corrales P. Fernandez F. Simoes M. R. Pecanha F. M.. (2017a). Egg white-derived peptides prevent cardiovascular disorders induced by mercury in rats: role of angiotensin-converting enzyme (ACE) and NADPH oxidase. Toxicol. Lett. 281, 158–174. 10.1016/j.toxlet.2017.10.001, PMID: 28987480

[ref42] Rizzetti D. A. Martinez C. S. Escobar A. G. da Silva T. M. Uranga-Ocio J. A. Pecanha F. M.. (2017b). Egg white-derived peptides prevent male reproductive dysfunction induced by mercury in rats. Food Chem. Ttoxicol. 100, 253–264. 10.1016/j.fct.2016.12.038, PMID: 28043836

[ref43] Rizzetti D. A. Torres J. G. Escobar A. G. da Silva T. M. Moraes P. Z. Hernanz R.. (2017c). The cessation of the long-term exposure to low doses of mercury ameliorates the increase in systolic blood pressure and vascular damage in rats. Environ. Res. 155, 182–192. 10.1016/j.envres.2017.02.022, PMID: 28222365

[ref44] Serafini M. Peluso I. (2016). Functional foods for health: the interrelated antioxidant and anti-inflammatory role of fruits, vegetables, herbs, spices and cocoa in humans. Curr. Pharm. Des. 22, 6701–6715. 10.2174/1381612823666161123094235, PMID: 27881064 PMC5427773

[ref45] Su L. Wang M. Yin S. T. Wang H. L. Chen L. Sun L. G.. (2008). The interaction of selenium and mercury in the accumulations and oxidative stress of rat tissues. Ecotoxicol. Environ. Saf. 70, 483–489. 10.1016/j.ecoenv.2007.05.018, PMID: 17644179

[ref46] Szasz T. Thakali K. Fink G. D. Watts S. W. (2007). A comparison of arteries and veins in oxidative stress: producers, destroyers, function, and disease. Exp. Biol. Med. 232, 27–37. PMID: 17202583

[ref47] Touyz R. M. Briones A. M. (2011). Reactive oxygen species and vascular biology: implications in human hypertension. Hypertens. Res. 34, 5–14. 10.1038/hr.2010.201, PMID: 20981034

[ref48] Valko M. Rhodes C. J. Moncol J. Izakovic M. Mazur M. (2006). Free radicals, metals and antioxidants in oxidative stress-induced cancer. Chem. Biol. Interact. 160, 1–40. 10.1016/j.cbi.2005.12.009, PMID: 16430879

[ref49] Vassallo D. V. Moreira C. M. Oliveira E. M. Bertollo D. M. Veloso T. C. (1999). Effects of mercury on the isolated heart muscle are prevented by DTT and cysteine. Toxicol. Appl. Pharmacol. 156, 113–118. 10.1006/taap.1999.8636, PMID: 10198276

[ref50] Virtanen J. K. Rissanen T. H. Voutilainen S. Tuomainen T. P. (2007). Mercury as a risk factor for cardiovascular diseases. J. Nutr. Biochem. 18, 75–85. 10.1016/j.jnutbio.2006.05.001, PMID: 16781863

[ref51] Wiggers G. A. Pecanha F. M. Briones A. M. Perez-Giron J. V. Miguel M. Vassallo D. V.. (2008). Low mercury concentrations cause oxidative stress and endothelial dysfunction in conductance and resistance arteries. Am. J. Physiol. Heart Circ. Physiol. 295, H1033–H1043. 10.1152/ajpheart.00430.2008, PMID: 18599595

