# Supplementary figures and images for: Antioxidant Properties of Egg White Hydrolysate Prevent Mercury-Induced Vascular Damage in Resistance Arteries
Source: Front Physiol. 2020 Nov 20;11:595767. doi: 10.3389/fphys.2020.595767 (PMC7714919; doi:10.3389/fphys.2020.595767)

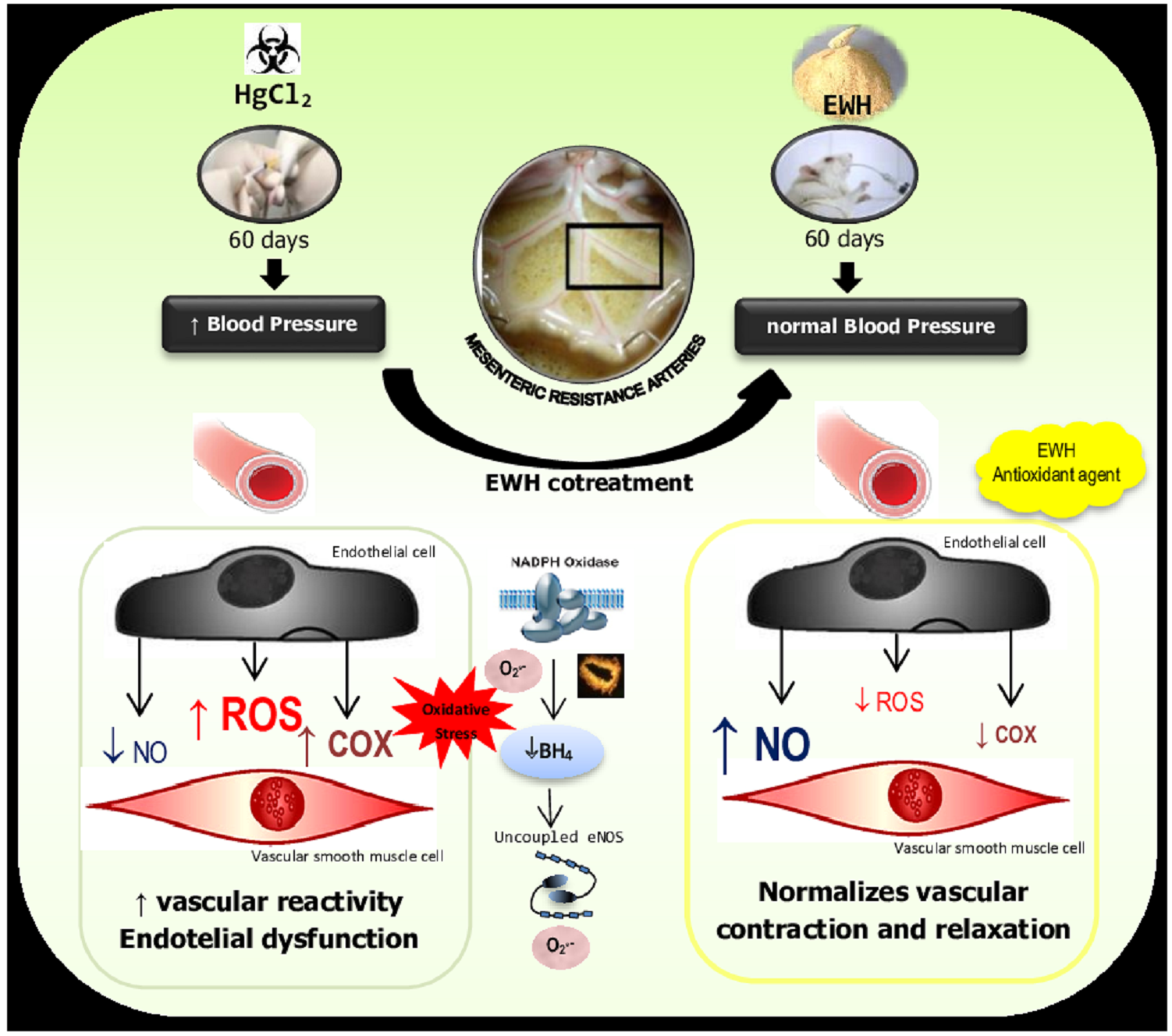

Supplement: Supplementary file 1 [file Image_1.tif]
